# Schwann Cell Precursors Generate the Majority of Chromaffin Cells in Zuckerkandl Organ and Some Sympathetic Neurons in Paraganglia

**DOI:** 10.3389/fnmol.2019.00006

**Published:** 2019-01-25

**Authors:** Maria Eleni Kastriti, Polina Kameneva, Dmitry Kamenev, Viacheslav Dyachuk, Alessandro Furlan, Marek Hampl, Fatima Memic, Ulrika Marklund, Francois Lallemend, Saida Hadjab, Laura Calvo-Enrique, Patrik Ernfors, Kaj Fried, Igor Adameyko

**Affiliations:** ^1^Department of Physiology and Pharmacology, Karolinska Institutet, Stockholm, Sweden; ^2^Center for Brain Research, Medical University of Vienna, Vienna, Austria; ^3^National Scientific Center of Marine Biology, Far Eastern Branch, Russian Academy of Sciences, Vladivostok, Russia; ^4^Department of Neuroscience, Karolinska Institutet, Stockholm, Sweden; ^5^Cold Spring Harbor Laboratory, Cold Spring Harbor, NY, United States; ^6^Institute of Animal Physiology and Genetics, CAS, Brno, Czechia; ^7^Department of Experimental Biology, Faculty of Science, Masaryk University, Brno, Czechia; ^8^Unit of Molecular Neurobiology, Department of Medical Biochemistry and Biophysics, Karolinska Institutet, Stockholm, Sweden

**Keywords:** Zuckerkandl organ, extra-adrenal chromaffin cells, posterior trunk sympathetic ganglia, para-aortic sympathetic ganglia, catecholamines, Schwann cell precursors

## Abstract

In humans, neurosecretory chromaffin cells control a number of important bodily functions, including those related to stress response. Chromaffin cells appear as a distinct cell type at the beginning of midgestation and are the main cellular source of adrenalin and noradrenalin released into the blood stream. In mammals, two different chromaffin organs emerge at a close distance to each other, the adrenal gland and Zuckerkandl organ (ZO). These two structures are found in close proximity to the kidneys and dorsal aorta, in a region where paraganglioma, pheochromocytoma and neuroblastoma originate in the majority of clinical cases. Recent studies showed that the chromaffin cells comprising the adrenal medulla are largely derived from nerve-associated multipotent Schwann cell precursors (SCPs) arriving at the adrenal anlage with the preganglionic nerve fibers, whereas the migratory neural crest cells provide only minor contribution. However, the embryonic origin of the ZO, which differs from the adrenal medulla in a number of aspects, has not been studied in detail. The ZO is composed of chromaffin cells in direct contact with the dorsal aorta and the intraperitoneal cavity and disappears through an autophagy-mediated mechanism after birth. In contrast, the adrenal medulla remains throughout the entire life and furthermore, is covered by the adrenal cortex. Using a combination of lineage tracing strategies with nerve- and cell type-specific ablations, we reveal that the ZO is largely SCP-derived and forms in synchrony with progressively increasing innervation. Moreover, the ZO develops hand-in-hand with the adjacent sympathetic ganglia that coalesce around the dorsal aorta. Finally, we were able to provide evidence for a SCP-contribution to a small but significant proportion of sympathetic neurons of the posterior paraganglia. Thus, this cellular source complements the neural crest, which acts as a main source of sympathetic neurons. Our discovery of a nerve-dependent origin of chromaffin cells and some sympathoblasts may help to understand the origin of pheochromocytoma, paraganglioma and neuroblastoma, all of which are currently thought to be derived from the neural crest or committed sympathoadrenal precursors.

## Introduction

Chromaffin cells are the main cell type responsible for the production and systemic release of circulating catecholamines. These include adrenaline and noradrenaline and allow the body to respond to stress induced by a variety of external stimuli. This stress-response is orchestrated by the cholinergic preganglionic neurons of the central nervous system, which directly control catecholamine release by peripheral neurosecretory chromaffin cells. Additionally, catecholamines are fundamental during embryonic development, participating in normal cardiac development, as well as in hypoxia-induced stress response *in utero* (Kobayashi et al., 1995; Thomas et al., 1995; Zhou et al., 1995; Rios et al., 1999; Portbury et al., 2003; Ream et al., 2008).

Even though the most well-known hub of chromaffin cells in mammals is the medulla of the adrenal gland, an additional chromaffin organ can be found next to the dorsal aorta, around the mid-level of the kidneys and in a close association with numerous sympathetic ganglia. This chromaffin organ, known as Zuckerkandl organ (ZO), is the largest extra-adrenal chromaffin body in mammals (Coupland, 1965; Böck, 1982; Zuckerkandl, 1901; Kohn, 1903). In rodents and other small mammals, ZO is a transient embryonic organ, which reaches maximal cell numbers just before or after birth and undergoes autophagy-mediated cell death, which is initiated in early postnatal stages (Schober et al., 2013). In humans, the ZO reaches the peak of its size around the 3rd year of life and then slowly regresses, thus timing of ZO disappearance is species-specific.

The connection between sympathetic neurons and chromaffin cells is not only functional, which is the case in stress-responses, but also has been rendered to be ontogenetic. Until recently, research supported the conclusion that during early embryogenesis multipotent neural crest cells migrate toward the dorsal aorta in two waves, and in turn differentiate toward either sympathetic or chromaffin cells as a response to secreted factors from the aorta (Huber et al., 2009; Saito et al., 2012). However, several studies have lately challenged that idea. Firstly, the progenitors of the two systems seem to express discrete markers even before they reach the area of the dorsal aorta (Ernsberger et al., 2005; Chan et al., 2016). Furthermore, recent studies proved that the two cell types are of different origin, with the majority of adrenal chromaffin cells being derived late from nerve-associated multipotent cells, also known as Schwann cell precursors (SCPs), which use the axons of the preganglionic neurons as a pathway to the sympathoadrenal (SA) anlage (Furlan et al., 2017; Lumb et al., 2018). Additionally, single-cell transcriptomic analysis of the developing SA progenitors allowed sampling of both sympathoblasts and chromaffin cells during early development and resulted in significant differences, as well as similarities, in the molecular profiles and markers of the two populations (Furlan et al., 2016, 2017).

The ZO, adrenal medulla and sympathetic ganglia are the locations of paraganglioma (PGG) and pheochromocytoma (PCC) (Huber et al., 2018). These tumors are very heterogeneous and their origin is still not fully understood, although they are considered to be composed of chromaffin cells (Lenders et al., 2014). Given their similarities and common features, it is becoming increasingly clear that it is important to fully understand the normal development of chromaffin and sympathetic structures found in the trunk.

In this study, using lineage tracing and genetic manipulations during embryonic mouse development, we revealed that the majority of chromaffin cells of the ZO are SCP-derived similarly to those of the adrenal medulla, in contrast to the majority of the cells of the sympathetic ganglia that are formed around the dorsal aorta from the migratory neural crest cells. At the same time, we identified a minor portion of SCP-derived sympathetic neurons in the posterior trunk paraganglia.

These findings reveal the difference in origin of sympathetic (mainly derived from neural crest cells) paraganglia and chromaffin cells of ZO (mainly derived from SCPs). Taking into account the role of innervation in the development of adrenal medulla and ZO organ, the SCP-dependent alternative origin of a minority of sympathetic neurons evokes a need to reconsider the potential cellular origin of different subtypes of neuroblastoma, PGG and PCC.

## Results

### Chromaffin Cells of Zuckerkandl Organ and Sympathoblasts of the Mid-to-Lower-Trunk Arise From Different Cellular Origins

**FIGURE 1:**
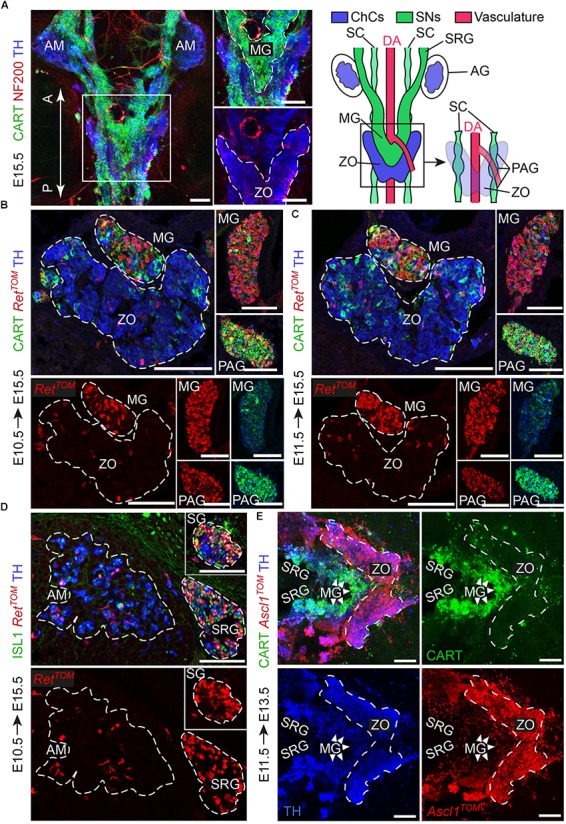
The sympathetic ganglia at the level of the Zuckerkandl organ and the organ itself have distinct early-defined origin despite the intermingling anatomy. **(A)** Dorsal view of whole-mount immunofluorescence (left panel) against the sympathetic marker CART, the chromaffin and sympathetic marker TH and NF200 (showing the innervation on the trunk of an E15.5 wild type embryo and schematic (right panel) showing the sympathetic and chromaffin structures in relation to the dorsal aorta. Note that the mesenteric (MG) and suprarenal ganglion (SRG), as well as the sympathetic chain (SC), are CART^+^, while the Zuckerkandl organ (ZO) is composed mainly by TH^+^/CART^-^ cells. Additionally note that the para-aortic ganglia (PAG) are the continuation of the sympathetic chain that extends along the anteroposterior axis of the embryo trunk just at the dorsal view of the dorsal aorta, at the level of the ZO and MG. **(B,C)** Immunofluorescence on cryosections against CART, *Ret*^TOM^ and TH on tamoxifen-injected (TAM-injected) embryos at E10.5 and E11.5 respectively shows that *Ret*^TOM^ specifically delineated the sympathetic compartment when analyzed at E15.5, with clear tracing of the MG and PAG, while only few *Ret*^TOM+^ cells can be seen in the ZO. Note the difference in CART immunofluorescence levels in the MG and PAG. **(D)** Immunofluorescence on cryosections against ISL1, *Ret*^TOM^ and TH on TAM-injected embryos at E10.5 shows *Ret*^TOM^ specific expression by the sympathetic ganglion (SG) and SRG when analyzed at E15.5, while almost no *Ret*^TOM+^ cells can be seen in the adrenal medulla (AM). **(E)** Ventral view of whole-mount immunofluorescence against CART, *Ascl1*^TOM^ and TH on embryos with TAM injection at E11.5 and analyzed at E13.5 shows tracing in the chromaffin cells of the ZO, while no tracing in the MG (shown by white arrowheads). Scale bar in **(A–E)** = 100 μm. A, anterior; P, posterior; AM, adrenal medulla; MG, mesenteric ganglion; ZO, Zuckerkandl organ; DA, dorsal aorta; SC, sympathetic chain; SRG, suprarenal ganglion; AG, adrenal gland; PAG, para-aortic ganglion; SG, sympathetic ganglion; ChCs, chromaffin cells; SNs, sympathetic neurons.)

Sympathetic neurons of the autonomic nervous system and neuroendocrine chromaffin cells share many similarities, such as expression of DBH and TH – enzymes involved in catecholamine synthesis (Huber, 2006). However, a detailed study during the development of the SA system showed that the two cell types can be resolved by means of CART and TH expression. Specifically, CART levels are high in newly formed sympathetic neurons, while TH levels are low. In contrast, in chromaffin cells, TH levels are high and CART is almost absent, even though a very small portion of chromaffin cells is CART^+^ (Chan et al., 2016). When it comes to the Zuckerkandl organ (ZO) and adrenal medulla (AM), the two chromaffin organs do not appear to have any molecular differences, but only anatomical and morphological discrepancies. As opposed to the chromaffin cells of the adrenal medulla, which are clearly separated from the sympathetic ganglia in their vicinity by the surrounding adrenal cortex, chromaffin cells of ZO are immediately surrounded by numerous sympathetic ganglia (Figures 1A,B). Additionally, whole mount immunofluorescence at E15.5 shows that the posterior part of the ZO is composed in a significant degree of CART^+^/TH^+^ cellular populations, hinting to a potential composite nature (Figure 1A).

From the literature, it is clear that the adrenal primordium can be identified as early as E11.5, but the first TH^+^ chromaffin cells in the area can only be identified a day later (Huber et al., 2009; Lumb et al., 2018). Thus, we chose to analyze the progenitors of the sympathetic and chromaffin lineages at the level of the ZO at E10.5 and E11.5.

During development, the receptor RET is expressed in a dynamic way by sympathetic neurons and chromaffin cells and is essential for the development of all sympathetic neurons, whereas it is dispensable for the development of the chromaffin cells of the adrenal medulla (Enomoto et al., 2001; Allmendinger et al., 2003). To test RET involvement in the lineage separation within the ZO and the adjacent sympathetic ganglia, we used the inducible *Ret*^CreERT2^;*R26*^TOM^ reporter line (referred from now on as *Ret*^TOM^). By injecting tamoxifen (TAM) at E10.5 or E11.5 and analyzing the ZO with the mesenteric/para-aortic sympathetic ganglia around it, we found that the two components are of different origin, with *Ret*^TOM+^ cells traced from either E10.5 or E11.5 giving rise only to the sympathetic mesenteric and para-aortic ganglia and not chromaffin cells of the ZO (Figures 1B,C). Thus, the lineages of sympathetic ganglia and chromaffin cells at the level of the ZO are already separated at E10.5, similarly to what was observed in the case of the sympathetic ganglia at the vicinity of the adrenal gland and the chromaffin cells of the adrenal medulla (Figure 1D) (Furlan et al., 2017). Additionally, we observed that the sympathetic identity-specific marker CART (Chan et al., 2016; Furlan et al., 2017) is expressed in lower levels in the mesenteric ganglion in comparison to the para-aortic ganglia in the vicinity of the ZO at E15.5 (Figures 1B,C, insets). Higher levels of CART expression in sympathetic neurons have been correlated with earlier developmental points, showing the peak of expression at E12.5, while CART is expressed in progressively lower levels at later stages (Chan et al., 2016). This suggests that the mesenteric ganglia are generated earlier or mature faster as compared to the para-aortic ganglia.

Another key gene that is important for the development of both sympathetic neurons and the medullary chromaffin cells is *Ascl1* (*Mash1*) (Huber et al., 2002a,b). Additionally, *Ascl1* is a master regulator transcription factor with dynamic expression during SA development, delineating only chromaffin cells when lineage tracing is initiated after E11.5 (Furlan et al., 2017). Using the inducible *Ascl1*^CreERT2^;*R26*^TOM^ reporter line, we injected TAM at E11.5. Analysis of *Ascl1*^TOM+^ embryos at E13.5 showed that the ZO is derived from E11.5 *Ascl1+* progenitors, while the mesenteric and other paraganglia are not derived from the *Ascl1*^TOM+^ lineage (Figure 1E), confirming the two separate lineages of the sympathetic neurons and chromaffin cells at the level of the ZO that we observed with the *Ret*^TOM^^+^ tracing.

Thus, by means of the inducible expression of *Ret*^TOM^^+^ and *Ascl1*^TOM^^+^, we showed that even though the sympathetic ganglia surrounding the ZO and the chromaffin cells of the ZO develop in a tight spatial association with each other, they are derived from different progenitors, as opposed to what was believed before.

### Zuckerkandl Organ and a Newly Identified Extra-Adrenal Chromaffin Body Are Largely Generated From Nerve-Derived SCPs

As mentioned above, cells of the *Ret*^+^ lineage traced from E10.5 and E11.5 delineated specifically the sympathetic mesenteric and para-aortic ganglia and not the chromaffin cells of ZO, indicating that the neuroendocrine chromaffin cells of the ZO are not derived from *Ret*^+^ precursors. It has been previously established that at E11.5 there are no more freely-migrating neural crest cells, and that only nerve-associated SCPs can be found in the mouse trunk (Furlan et al., 2017). Thus, given the absence of *Ret*^TOM+^ cells traced from E10.5 and E11.5 in the chromaffin cells of the ZO, we reasoned that the organ could be derived from nerve-associated SCPs, similarly to the chromaffin cells of the adrenal gland (Furlan et al., 2017).

**FIGURE 2:**
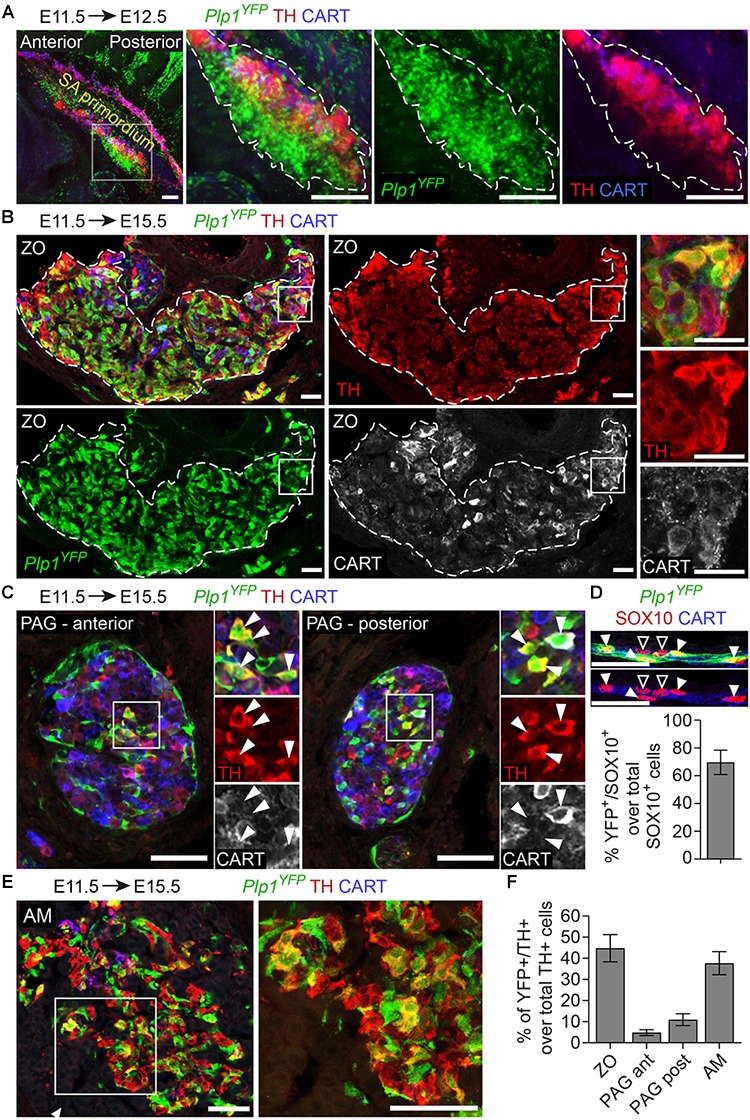
The chromaffin cells of the Zuckerkandl organ are grossly Schwann-cell-precursor-derived, as well as a small portion of the posterior trunk sympathetic ganglia. **(A)** Side view of whole-mount immunofluorescence against *Plp1*^YFP^, TH and CART on an E12.5 embryo which received a single tamoxifen (TAM) injection at E11.5, showing the sympathoadrenal (SA) primordium area. Note the big accumulation of *Plp1*^YFP+^ cells ventrally to the first TH^+^ cells and the lack of *Plp1*^YFP+^ cells at the more overlying CART^+^ chain corresponding to the sympathoblasts of the sympathetic chain. **(B,C,E)** Immunofluorescence on cryosections against *Plp1*^YFP^, CART and TH on an E15.5 embryo injected with TAM at E11.5 and analyzed at E15.5. Note the *Plp1*^YFP+^/TH^+^ cells in the Zuckerkandl organ (ZO), sympathetic para-aortic ganglion (PAG, shown by white arrowheads) and adrenal medulla (AM). **(D)** Upper panel: immunofluorescence against *Plp1*^YFP^, SOX10 and CART on peripheral nerves of E15.5 *Plp1*^YFP+^ embryos injected with TAM at E11.5. Lower: quantification of recombination efficiency in SOX10^+^ cells of peripheral nerves (corresponding to total peripheral glial cells) of embryos TAM-injected on E11.5 and analyzed at E15.5 (69.63 ± 8.69%, *N* = 4). Filled arrowheads show *Plp1*^YFP+^/SOX10^+^ glial cells and empty arrowheads show *Plp1*^YFP-^/SOX10^+^ glial cells. **(F)** Quantification of recombination in chromaffin and sympathetic population in embryos TAM-injected on E11.5 and analyzed at E15.5 (for the ZO 44.77 ± 6.44%, anterior PAG 4.84 ± 1.38%, posterior PAG 11 ± 2.80%, AM 37.69 ± 5.46%, in all cases *N* = 4). Data are presented as mean ± SEM. Note the high percentage of *Plp1*^YFP+^/TH^+^ cells in the chromaffin organs ZO and AM and the difference in percentage of *Plp1*^YFP+^/TH^+^ cells between the posterior and anterior PAG. Scale bar in **(A)** = 100 μm, in **(B–E)** = 50 μm. SA primordium, sympathoadrenal primordium; ZO, Zuckerkandl organ; PAG (ant), para-aortic ganglion (anterior); PAG (post), para-aortic ganglion (posterior); AM, adrenal medulla.

**FIGURE 3:**
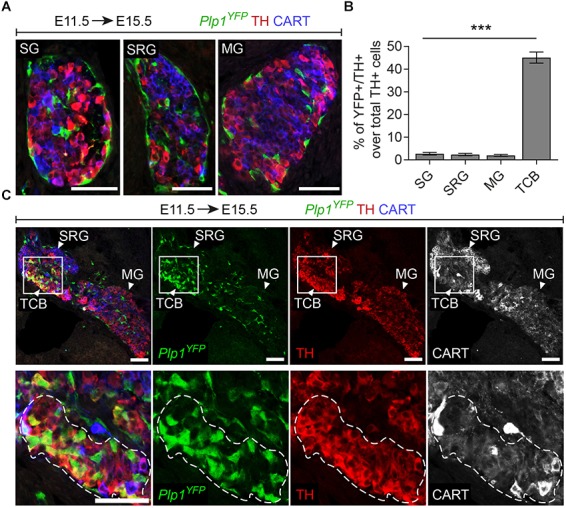
A chromaffin body located between the suprarenal and mesenteric ganglion is Schwann-cell-precursor-derived. **(A)** Immunofluorescence on cryosections against *Plp1*^YFP^, CART and TH on an E15.5 embryo injected with tamoxifen (TAM) at E11.5 showing the sympathetic ganglion (SG), suprarenal ganglion (SRG) and mesenteric ganglion (MG). **(B)** Quantification of recombination in chromaffin and sympathetic population in embryos TAM-injected on E11.5 and analyzed at E15.5 (SG 2.72 ± 0.65%, SRG 2.35 ± 1.04%, MG 1.96 ± 0.81%, TCB 45.09 ± 4.90%, in all cases *N* = 4). Data are presented as mean ± SEM. In TCB vs. SG *P* = 0.0004, TCB vs. SRG *P* = 0.0003 and TCB vs. MG *P* = 0.0005. **(C)** Immunofluorescence on cryosections against *Plp1*^YFP^, CART and TH on an E15.5 embryo injected with TAM at E11.5 at the area of transition from the SRG to the MG, showing the “transitional chromaffin body” (TCB). Note the differential CART and TH signal between the SRG and MG, as well as the *Plp1*^YFP+^/TH^high^ cells in the TCB. Note the high percentage of *Plp1*^YFP+^/TH^+^ cells in the TCB and the absence of *Plp1*^YFP+^/TH^+^ cells in the SG, SRG and MG. Scale bar in **(A,C)** = 50 μm. SG, sympathetic ganglion; SRG, suprarenal ganglion; MG, mesenteric ganglion; TCB, transitional chromaffin body; ^∗∗∗^*P* ≤ 0.001.

To test this, we first used the *Plp1*^CreERT2^;*R26*^YFP^ reporter line (referred to from now on as *Plp1*^YFP^), a strain that has been used in the past to trace peripheral glia (or Schwann cells) during development and in adult mice (Leone et al., 2003; Goebbels et al., 2010; Adameyko et al., 2012; Dyachuk et al., 2014). It was previously shown that neural crest migration is complete by E11.5 and at this stage there are no neural crest cells at the embryo trunk which are multipotent and can give rise directly to sympathetic neurons (Furlan et al., 2017). Thus, in order to trace strictly SCPs and not neural crest cells, we injected TAM at E11.5. This resulted in tracing of SCPs along the nerves exiting the spinal cord and at the vicinity of the SA primordium by E12.5 (Figure 2A). TAM injection at E11.5 and analysis of the ZO at E15.5 revealed that SCPs contributed by 44.7 ± 6.44% to these extra-adrenal chromaffin cells (Figures 2B,F). Importantly, we also observed a small, but significant percentage (11 ± 2.8% of the posterior para-aortic ganglionic neurons) of sympathetic neurons at the posterior levels of the trunk being traced (Figures 2C,F). In accordance to previously described SCP-contribution to the adrenal medulla (Furlan et al., 2017), 37.69 ± 5.47% of the adrenal medulla cells were traced, while there was no tracing of the sympathetic neurons of the anterior sympathetic ganglia and suprarenal ganglion (SRG) or mesenteric ganglion (<2.8% of the total sympathoblasts that compose the ganglia, Figures 2E,F, 3A,B). Taking in consideration that the efficiency of recombination in the total Schwann cell population along the nerves as seen by SOX10^+^ immunoreactivity (Kuhlbrodt et al., 1998; Britsch et al., 2001) was 69.63 ± 8.70% (Figure 2D), the total contribution of SCPs to the sympathetic neurons of the posterior trunk might be even greater.

Additionally, in *Plp1*^YFP+^ embryos traced from E11.5 and analyzed at E15.5, we consistently observed a group of TH^+^ cells, located in the transition between the SRG and mesenteric ganglion (Figure 3C). We termed this new cellular entity as “transitional chromaffin body” (TCB). Even though the TCB contained few TH^high^/CART^+^ cells in addition to the majority of TH^high^/CART^-^ cells, we excluded the possibility of this group of cells being a sympathetic ganglion, since it was composed of TH^high^ cells, typically a feature of early chromaffin cells and not sympathoblasts (as compared to the CART^high^/TH^low^ cells of the SRG and CART^low^/TH^low^ cells of the mesenteric ganglion, Figure 3C) (Chan et al., 2016). At the same time, almost half the cells of the TCB were *Plp1*^YFP+^ when traced starting from E11.5 (45.08% of the total cells), indicating that they are SCP-derived (Figures 3B,C). Interestingly, observation of the surrounding sympathetic neurons of the SRG and mesenteric ganglion revealed significant higher CART levels in the SRG compared to the mesenteric ganglion (Figure 3C).

**FIGURE 4:**
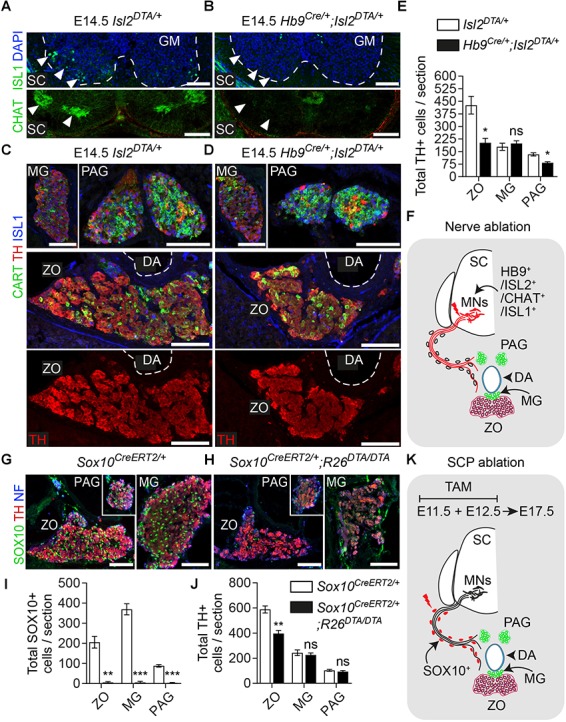
Ablation of Schwann cell precursors or the preganglionic nerves that serve as their route toward the dorsal aorta results in a reduction of chromaffin cell numbers of the Zuckerkandl organ. **(A,B)** Immunofluorescence on cryosections against CHAT (choline acetyltransferase) (upper panels) at the level of the spinal cord shows the absence of CHAT^+^ (cholinergic) preganglionic motorneurons in the gray matter, also seen as absence of ISL1^+^ motorneurons (lower panel). Scale bar = 100 μm. **(C,D)** Immunofluorescence against CART and TH at the level of the Zuckerkandl organ (ZO) shows a severely abnormal phenotype in the ZO and para-aortic ganglia (PAG) in *Hb9*^Cre/+^;*Isl2*^DTA/+^ E14.5 embryos in comparison to *Isl2*^DTA/+^ control E14.5 embryos. Scale bar = 100 μm. **(E)** Quantification of TH^+^ cells at E14.5 in control *Isl2*^DTA/+^ and mutant *Hb9^Cre/+^;*Isl2**^DTA/+^ embryos shows a decrease in the ZO and PAG, while the mesenteric ganglia (MG) remain unaffected. In *Isl2*^DTA/+^ versus *Hb9^Cre/+^;*Isl2**^DTA/+^ respectively: total TH^+^ cells in ZO = 426.6 ± 52.66 vs. 201.9 ± 27.43 (*P* = 0.0194), in MG = 178.4 ± 21.44 vs. 198.4 ± 15.34 (*P* = 0.4889) and in PAG = 133.1 ± 9.57 vs. 83.22 ± 5.79 (*P* = 0.0112), *N* = 3 per genotype. Data are presented as mean ± SEM and statistical analysis was performed using two-tailed Student *t*-test. **(F)** Schematic showing the experimental design for induction of the visceral nerve ablation using the *Hb9*^Cre^;*Isl2*^DTA^ strain. **(G,H)** Immunofluorescence against SOX10, TH and neurofilaments (NF) on cryosections from *Sox10*^CreERT2/+^ and *Sox10*^CreERT2/+^;*R26*^DTA/DTA^ E17.5 embryos following tamoxifen (TAM) injection at E11.5 and E12.5 and analysis at E17.5 showing almost complete Schwann cell precursor (SCP)-ablation and significantly abnormal morphology in ZO, PAG and MG. Scale bar = 100 μm. **(I)** Quantification of SOX10^+^ cells in control *Sox10*^CreERT2/+^ and SCP-ablated *Sox10*^CreERT2/+^;*R26*^DTA/DTA^ E17.5 embryos following TAM injection at E11.5 and E12.5 and analysis at E17.5. In *Sox10*^CreERT2/+^ vs. *Sox10*^CreERT2/+^;*R26*^DTA/DTA^ respectively: total SOX10^+^ cells in ZO = 205.47 ± 28.96 vs. 5.33 ± 4.43 (*P* = 0.0024), in MG = 369.33 ± 27.56 vs. 5.55 ± 5.22 (*P* = 0.0002) and in PAG = 88.11 ± 6.46 vs. 3.24 ± 2.58 (*P* = 0.0002), *N* = 3 per genotype. Data are presented as mean ± SEM and statistical analysis was performed using two-tailed Student *t*-test. **(J)** Quantification of TH+ cells in control *Sox10*^CreERT2/+^ and SCP-ablated *Sox10*^CreERT2/+^;*R26*^DTA/DTA^ E17.5 embryos following TAM injection at E11.5 and E12.5 and analysis at E17.5. In *Sox10*^CreERT2/+^ vs. *Sox10*^CreERT2/+^;*R26*^DTA/DTA^ respectively: total TH^+^ cells in ZO = 588.08 ± 28.41 vs. 397.22 ± 23.69 (*P* = 0.0067), in MG = 243.66 ± 22.98 vs. 226.55 ± 15.24 (*P* = 0.5685) and in PAG = 103.00 ± 7.61 vs. 94.11 ± 8.04 (*P* = 0.4672), *N* = 3 per genotype. Data are presented as mean ± SEM and statistical analysis was performed using two-tailed Student *t*-test. **(K)** Schematic outlining the TAM injection times and collection time point in the Schwann cell precursor (SCP)-ablation experiment using the *Sox10*^CreERT2^;*R26*^DTA^ strain. SC, spinal cord; GM, gray matter; MG, mesenteric ganglion; PAG, para-aortic ganglion; MNs, motorneurons; DA, dorsal aorta; ZO, Zuckerkandl organ; ns, non-significant; ^∗^*P* ≤ 0.05; ^∗∗^*P* ≤ 0.01; ^∗∗∗^*P* ≤ 0.001.

The adrenal gland receives innervation from the cholinergic (neurons positive for choline acetyltransferase – CHAT^+^) preganglionic neurons of the spinal cord (Parker et al., 1993). Due to the compactness of the first TH^+^ chromaffin cells (or else SA primordium) (Figure 2A), we reasoned that potentially the same fibers innervate the ZO. In order to test this and to further confirm that the *Plp1*^YFP+^ cells, which contributed to ZO and paraganglia, were nerve-associated SCPs, we used a genetic model for specific ablation of these nerves. To achieve this, we utilized the *Hb9*^Cre^;*Isl2*^DTA^ strain. In this strain, *Hb9*^+^ preganglionic visceral neurons start express *Isl2*-driven DTA (Diptheria Toxin Subunit A) as soon as they exit cell cycle, resulting to their death (Yang et al., 2001; Thaler et al., 2004; Furlan et al., 2017) (Figures 4A,B,F). As a result, the nerves that serve as routes for SCPs to arrive at the SA anlage are ablated to a significant extent. Upon loss of the preganglionic motorneurons and analysis at E14.5, we observed a significant reduction of the total TH^+^ cells of the ZO (52.72% reduction) and para-aortic sympathetic ganglia (37.47% reduction), while the mesenteric ganglia remained unaffected (Figures 4C–E).

Next, to exclude any potential secondary effect of the nerve ablation on the development of the ZO due to the lack of trophic factors produced by the nerve, we performed SCP-ablation experiments by means of the *Sox10*^CreERT2^;*R26*^DTA^ strain. To induce a significant decrease in SCPs (all of which are SOX10+), we injected TAM both at E11.5 and E12.5 (Figure 4K). Analysis of the SCP-ablated embryos was performed at E17.5 and showed a significant loss of glial (SCP) SOX10^+^ cells in ZO and the surrounding mesenteric and para-aortic sympathetic ganglia (Figures 4G–J). Upon SCP-ablation, ZO was significantly affected, as shown by a 32.45% decrease in total TH^+^ cell numbers. However, even though an abnormal morphology was observed for the mesenteric and para-aortic ganglia, the changes in TH^+^ numbers and the overall reduction of these two sympathetic structures were not statistically significant (Figures 4G–J).

Thus, both types of experimental approaches of blocking the delivery of SCPs to the SA anlage (either by ablating the delivering nerve or by ablating the SCPs themselves) demonstrated an effect on ZO chromaffin and possibly sympathetic cells and no significant effect on other sympathetic ganglia of the region.

The current belief supports that the neurons of the sympathetic nervous system are derived from the neural crest. However, a variety of observations hint that at least a small proportion of the sympathetic neurons at the posterior trunk are derived from SCPs. Firstly, upon TAM injection at E11.5 in *Plp1*^YFP^ mice, we find a 5–10% tracing of the sympathetic neurons of the para-aortic ganglia. Secondly, we observe a noticeable reduction in the size of the para-aortic ganglia upon ablation of the visceral motorneurons, which are responsible for the delivery of SCPs to the SA anlage. However, glial ablation by TAM injection at E11.5 and E12.5 does not seem to significantly affect these ganglia, which could be due to the non-extensive expression of the transgene in this population.

### *Ascl1* Is Indispensable for SCP Differentiation to Chromaffin Cells of the ZO

Previous studies have highlighted the importance of *Ascl1* (also known as *Mash1*) in the transition of SCPs to mature neuroendocrine TH^+^ chromaffin cells residing in adrenal medulla (Huber et al., 2002a; Furlan et al., 2017). Additionally, the gene is also indispensable for the development of sympathetic ganglia of the autonomic nervous system.

The lineage tracing experiments proved that *Ascl1*^+^ progenitors contribute to the development of ZO (Figure 1D), and suggested that the absence of *Ascl1* should inhibit the differentiation of SCPs toward chromaffin cells in ZO. In order to examine this, we used the knock-in *Ascl1*^CreERT2^;*R26*^TOM^ strain, where the coding sequence of the *Ascl1* gene is replaced by *CreERT*. At the same time, both heterozygote and homozygote embryos express *Ascl1*^TOM^, which allows to track the progeny of the cells that lack *Ascl1*, in the case of the homozygosity.

**FIGURE 5:**
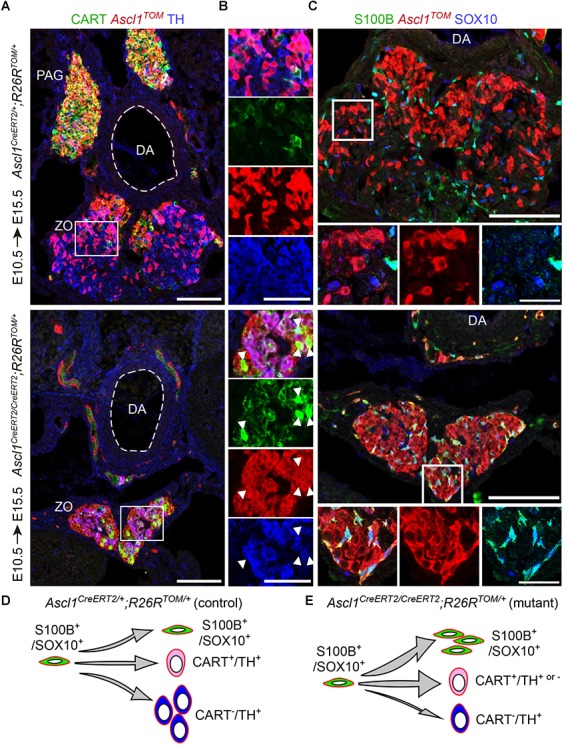
*Ascl1* is indispensable for differentiation of Schwann cell precursors toward chromaffin cells of the Zuckerkandl organ. **(A,B)** Immunofluorescence against CART, *Ascl1*^TOM^ and TH on cryosections of tamoxifen (TAM)-injected embryos at E10.5 and analyzed at E15.5 shows a decrease in TH^+^/*Ascl1*^TOM+^ cells in the region of the Zuckerkandl organ (ZO) in mutant *Ascl1*^CreERT2/CreERT2^;*R26*^TOM/+^ embryos in comparison to control *Ascl1*^CreERT2/+^;*R26*^TOM/+^ embryos, as well as increased CART^+^/*Ascl1*^TOM+^ cell numbers (shown by white arrowheads) and absence of the para-aortic ganglia (PAG). **(C)** Immunofluorescence against S100B, *Ascl1*^TOM^ and SOX10 on cryosections of TAM-injected embryos at E10.5 and analyzed at E15.5 shows an accumulation of S100B^+^/SOX10^+^/*Ascl1*^TOM+^ cells in the region of the ZO in mutant *Ascl1*^CreERT2/CreERT2^;*R26*^TOM/+^ embryos that are absent in control *Ascl1*^CreERT2/+^;*R26*^TOM/+^ embryos. Scale bar in **(A)** and in upper panel of **(C)** = 100 μm, in **(B)** = 50 μm and in magnified insets in **(C)** = 25 μm. **(D,E)** Schematic depicting the main findings upon *Ascl1* ablation and tracing of the *Ascl1*-progeny. The *Ascl1*^TOM^ lineage traced from E10.5 gives rise mainly to chromaffin cells (TH^+^) and sympathetic neurons (CART^+^) in control embryos. In contrast, in mutant embryos, the majority of *Ascl1*^TOM^ cells remains as glia (S100B^+^/SOX10^+^), with a small population of CART^+^ cells that is not seen in the control, and very few chromaffin cells (TH^+^) are produced. PAG, para-aortic ganglion; DA, dorsal aorta; ZO, Zuckerkandl organ; MG, mesenteric ganglion.

As expected, embryos which received TAM at E10.5 and were analyzed at E15.5, revealed tracing in the sympathetic compartment and a portion of the ZO chromaffin cells in heterozygous control embryos (*Ascl1*^CreERT2/+^;*R26*^TOM/+^) (Figure 5A). Contrary to this, homozygous mutant embryos (*Ascl1*^CreERT/CreERT2^;*R26*^TOM/+^) lacked the mesenteric ganglion and para-aortic sympathetic ganglia located above the dorsal aorta and featured an obvious decrease of TH^+^ chromaffin cells in ZO (Figures 5A,B). Furthermore, in the location of the mutant ZO, we observed TH^+^/CART^high^/*Ascl1*^TOM+^ and TH^-^/CART^high^/*Ascl1*^TOM+^ cells almost uniquely in mutant embryos, which might mean that *Ascl1* is also involved in the downregulation of sympathetic-like markers such as CART in immature progenitors in order for them to differentiate into mature TH^+^/CART^-^ cells, or that a very small portion of the mesenteric ganglion was formed but did not separate from the future ZO. In heterozygotes, few TH^-^/CART^+^/*Ascl1*^TOM+^ cells were observed only within the mesenteric ganglion, located just next to the dorsal aorta, above the ZO and were not included in the analysis of the ZO (Figure 5A). Furthermore, analysis of E15.5 embryos using the SCP (glial) markers S100B and SOX10 revealed an accumulation of S100B^+^/SOX10^+^/*Ascl1*^TOM+^ SCPs in mutant embryos, which were almost absent in control embryos (Figure 5C).

These results suggest that in the absence of *Ascl1*, the SCPs that serve as progenitors of the future chromaffin cells are not able to differentiate and are abnormally accumulating either in the SCP stage (as shown by the presence of S100B^+^/SOX10^+^/*Ascl1*^TOM+^ cells in the mutant) or in the transition from an immature neuroblast-like cell to a mature chromaffin cell (as seen by the numerous CART^+^/*Ascl1*^TOM+^ cells in the mutant within the abnormal ZO) (Figures 5D,E).

### Anatomical Complexity of ZO and Associated Sympathetic Paraganglia

**FIGURE 6:**
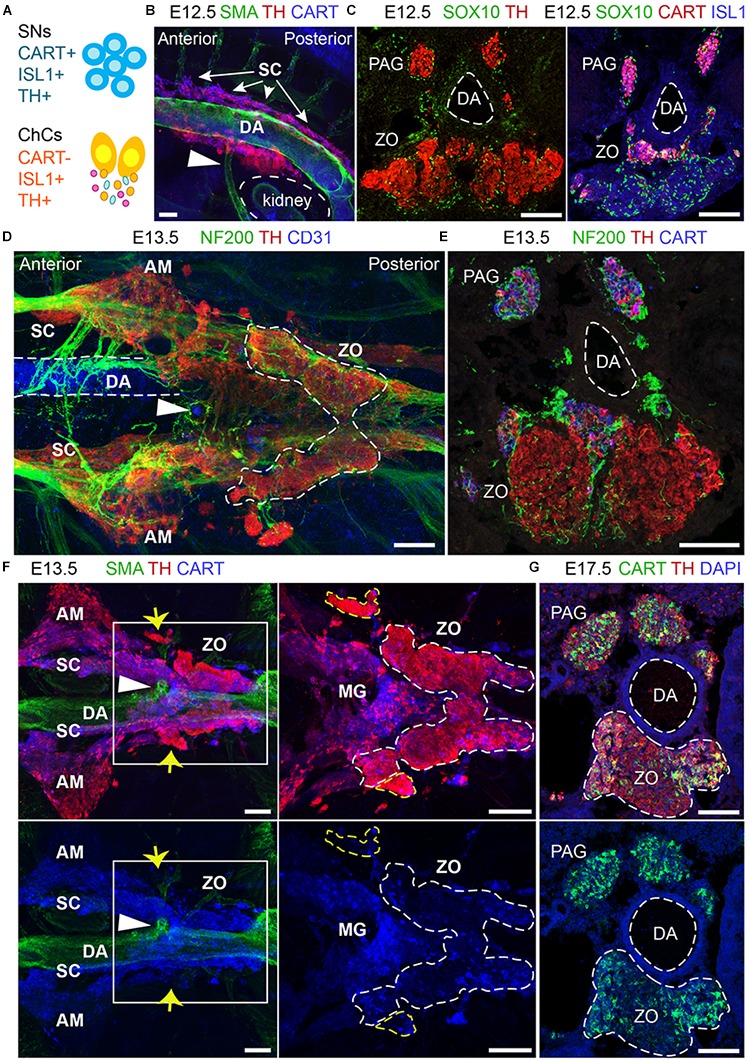
The development and anatomy of the Zuckerkandl organ and associated paraganglia in relation to the dorsal aorta. **(A)** Schematic showing the molecular differences and similarities between sympathetic neurons (SNs) and chromaffin cells (ChCs) during development in relation to the expression of CART, (ISL1 and TH. **(B)** Side-view of whole-mount immunofluorescence against smooth-muscle-actin (SMA, showing the dorsal aorta -DA), TH and CART on an E12.5 wild type embryo. Note the presence of TH^+^ cells at the dorsal part of the DA just posteriorly to its branching into the inferior mesenteric artery (shown by the white arrowhead). **(C)** Immunofluorescence on E12.5 wild type embryos against SOX10 and TH (left panel) and SOX10, CART and ISL1 (right panel) showing the close proximity of the developing CART^-^/ISL1^+^/TH^+^ Zuckerkandl organ (ZO) and CART^+^/ISL1^+^/TH^+^ mesenteric ganglion (MG). **(D)** Ventral-view of whole-mount immunofluorescence against NF200, TH and CD31 (showing the endothelium of the DA) on an E12.5 wild type embryo. Note the presence of the ZO below the inferior mesenteric artery (shown by the white arrowhead), which is surrounded by NF200^+^ axons. **(E)** Immunofluorescence on cryosection of an E13.5 wild type embryo against NF200, TH and CART. Note the innervation pattern around the DA and the TH+ cells of the ZO. **(F)** Ventral-view of whole-mount immunofluorescence against SMA, TH and CART on an E13.5 wild type embryo. Note the close proximity of the MG and ZO just posteriorly to the inferior mesenteric artery and the separation of the two structures based on the CART^+^/TH^low^ immunoreactivity in the MG in contrast to the TH^high^ immunoreactivity of the ZO (shown by the white arrowhead). Also note the presence of TH^high^ chromaffin structures in close proximity to the MG and ZO (shown by yellow arrows). **(G)** Immunofluorescence against CART, TH and DAPI on cryosections of a wild type E17.5 embryo, clearly showing the composite nature of the ZO at this later developmental stage, with both TH^+^/CART^-^ and TH^+^/CART^+^ cells. Scale bar = 100 μm. SNs, sympathetic neurons; ChCs, chromaffin cells; SC, sympathetic chain; DA, dorsal aorta; PAG, para-aortic ganglion; ZO, Zuckerkandl organ; AM, adrenal medulla; MG, mesenteric ganglion.)

The ZO is thought to be a chromaffin organ with similar properties to those of the adrenal medulla. However, the close association of the ZO with the dorsal aorta, its direct exposure to the environment due to the lack of a surrounding cortex, as well as its transient appearance as opposed to the adrenal medulla, suggest that it may have functions that have not been addressed. An element of complexity toward the study of the ZO is the fact that the main marker of chromaffin cells, TH, is also expressed by the vast majority of embryonic sympathetic neurons. Thus, only recent developments and discoveries have made it possible to study the ZO in a new light, such as single-cell data of the SA region during mid-gestation in mice and mature sympathetic neurons in adult mice (Furlan et al., 2016, 2017). The mentioned studies showed that the sympathoblasts are different from the chromaffin cells in terms of gene expression and brought to light markers that would discriminate between the two, such as CART, which was also supported by a previous study (Chan et al., 2016) (Figure 6A).

Even though our data show that the ZO and the AM share the SCP-origin, they also hint that the ZO might be a composite organ, as seen by CART^+^ immunoreactivity at the posterior part.

To reveal the developmental dynamics of ZO and in order to address whether the developing chromaffin component of the ZO associates with sympathetic structures while eventually integrating into them, we sought out to analyze extensively its development and location in relation to the sympathetic ganglia of the mid-trunk in various developmental stages.

Whole-mount immunofluorescence and immunofluorescence on cryosections using the sympathetic and chromaffin pan-marker TH and the sympathetic-specific marker CART at E12.5 placed the primordium of the developing ZO and adrenal medulla nearby sympathetic para-ganglia at the ventral part of the dorsal aorta (Figure 6B). At this stage, the two populations are in proximity and are found at the mid-level of the kidneys. We noticed that already at E12.5, a large population of CART^-^/TH^+^ cells is located below the branching of the dorsal aorta toward the inferior mesenteric artery (Figure 6B), clearly separated by the smaller CART^+^/TH^+^ population that corresponds to the sympathetic para-ganglia that have already been fate-defined (Figure 6C).

Additionally, whole-mount analysis of E13.5 embryos, as well as analysis on cryosections, revealed an elaborate innervation of the developing ZO (Figures 6D,E). Whole-mount immunostaining experiments at E13.5 demonstrated that the inferior mesenteric artery is not only surrounded by ZO (as shown by high TH+ immunofluorescence), but also by the mesenteric sympathetic ganglia (CART^+^/TH^low^), which previously might have been considered a part of the ZO at this stage (Figure 6F). Additionally, we also detected bilateral groups of CART^-^/TH^high^ cells that are not a part of ZO (Figure 6F, yellow arrows), and those cells have not been described in the literature before.

The complexity of the chromaffin and sympathetic structures of the mid-trunk becomes even more obvious upon examination of single cross-sections at the level of ZO at later developmental stages. The ZO is a continuously growing organ, reaching the maximum of chromaffin cell numbers just before birth in rodents (Schober et al., 2013). Notably, at E17.5, the sympathetic marker CART, which helped to differentiate between the sympathetic and chromaffin cells with high fidelity in earlier stages, shows immunoreactivity within the chromaffin population of ZO (Figure 6G). This observation might point to the hypothetical existence of several stages in the maturation of chromaffin cells that compose ZO or even hint that the organ might undergo fate reprogramming during late embryonic stages. Last but not least, these data suggest that ZO is not strictly a chromaffin population, but is likely to be a composite organ at the fullest of its maturity, consisting of chromaffin cells, undetermined bipotent sympatho-adrenal progenitors, peripheral glial cells and a fraction of sympathetic neurons.

Thus, our analysis shows for the first time the developmental complexity of the ZO, as well as the existence of a variety of chromaffin accumulations in its vicinity that do not seem to belong to ZO itself. Additionally, even though the ZO shares many similarities with the adrenal medulla, it is a more heterogeneous cellular population than the adrenal medulla, with a potential sympathetic-like component. Considering the classification of tumors that are found in the area of the ZO, our results are pointing to the possibility of a misclassification of some of these cancers. The existence of additional extra-adrenal chromaffin cellular accumulations, apart from the ZO, as well as the CART^+^ component of the ZO suggests that there are alternative cellular sources of PGG and PCC as those that were considered until now.

## Materials and Methods

### Mouse Strains and Husbandry

In all experiments, the day of plug detection was considered as E0.5. All experiments were performed under the permission of the Ethical Committee on Animal Experiments (Stockholm North committee) and according to The Swedish Animal Agency’s Provisions and Guidelines for Animal Experimentation recommendations.

#### Tamoxifen-Inducible CreERT2 Strains

*Sox10*^CreERT2^ were received from V. Pachnis laboratory (The Francis Crick Institute, United Kingdom) (MGI ID: 5301107). *Ret*^CreERT2^ mice were received from D. Ginty laboratory (Harvard University, United States) (MGI ID: 4437245). *Plp1*^CreERT2^ were received from U. Suter laboratory (ETH Zurich, Switzerland) (MGI ID: 2663093). *Ascl1*^CreERT2^ mice (a knock-in strain where the *Ascl1* coding region is replaced by the *CreERT2* sequence) were received from The Jackson Laboratory (stock 012882).

#### Cre-Dependent Reporter Strains

*R26R*^Tomato^ and *R26R*^YFP^ mice were ordered from The Jackson Laboratory (stock numbers 007914 and 006148 respectively).

All tracing experiments using inducible *CreERT2* lines (*Plp1, Sox10, Ret* and *Ascl1*-driven) were performed using heterozygotes for both the *CreERT2* and the reporter *R26R*^YFP^ or *R26R^TOMATO^*.

#### Strains Used in Cholinergic Preganglionic Motorneuron Ablation Experiments

For targeted ablation of the preganglionic neurons, *Isl2*^DTA^ mice were bred to *Hb9*^Cre^ mice to generate experimental *Hb9^Cre/+^;*Isl2**^DTA/+^ and control *Isl2*^DTA/+^ embryos.

*HB9*^Cre^ mice were received from The Jackson Laboratory (stock 006600). *Isl2*^DTA^ mice were received from The Jackson Laboratory (stock 007942).

#### Strains Used in Glia (SCP)-Ablation Experiments

For the glia ablation using the *Sox10*^CreERT2^;*R26*^DTA^ strain, all embryos were bred heterozygotes for the *Sox10*^CreERT2^ (V. Pachnis laboratory, The Francis Crick Institute, United Kingdom, MGI ID: 5301107) but homozygous for the *R26*^DTA^ (stock 006600).

#### Tamoxifen Dosage and Injection

To induce Cre-dependent recombination, tamoxifen (TAM) (Sigma, T5648) was dissolved freshly in corn oil (Sigma, Cat No 8267) as a 20 mg/mL stock and pregnant females received intraperitoneal (i.p.) injections during the desired embryonic stage (0.1 mg of TAM/g of body weight). When injecting at two time points, the total dose was divided into the course of 2 days.

### Embryo Collection and Preparation

Embryos were collected by evisceration from the uterus and a small incision was made at the region of the abdomen to allow penetration of the solutions. Then, the embryos were washed in ice-cold PBS (pH 7.4) and fixed in 4% paraformaldehyde (pH 7.4) in PBS at 4°C with mild rotation, with a total fixation duration adjusted to the developmental stage (3 h for E12.5 and E13.5 embryos, 5 h for E14.5 and E15.5 embryos, O/N for E17.5 embryos). Samples were washed in PBS three times at 4°C for 1 h in total.

In the case of desired collection of cryosections, the samples where cryoprotected by incubating at 4°C overnight in 30% sucrose in PBS until sinking. Next, tissue samples were embedded in OCT and frozen at -20°C.

In the case of whole mount immunofluorescence, the embryos were washed in PBS containing 0.1% Tween-20 (PBST) three times at room temperature, 10 min each time, before proceeding to the whole mount preparation.

### Immunofluorescence on Cryosections

Tissue samples were embedded in OCT and frozen at -20°C. Tissue was sectioned on the cross-sectional plane at 14–25 μm and frozen at -20°C after drying at room temperature for at least 1 h. Antigen retrieval was performed by incubation of the sections in 1x Target Retrieval Solution (Dako, S1699) for 20 min, pre-heated at 80°C. Sections were washed three times in PBST, 5 min each time, and incubated at 4°C overnight with primary antibodies diluted in PBST. Finally, sections were washed three times in PBST, 5 min each, and incubated with secondary antibodies diluted in PBST at room temperature for 1 h, washed three times in PBST and mounted using Mowiol mounting medium.

#### Primary Antibodies Used

Rabbit anti-TH (1:1000, Pel-Freez Biologicals, #P40101-150), sheep anti–TH (1:2000, Novus Biologicals, #NB300-110), mouse anti-TH (1:2000, Sigma, #T2928), mouse monoclonal anti-bIII tubulin (1:500, Promega, #G712A), goat anti-GFP (1:500, Abcam, #ab6662), chicken anti-GFP (1:500, Aves Labs Inc., #GFP-1020), chicken anti-mCherry (1:1000, EnCor Biotech, #CPCA-mCherry), goat anti-SOX10 (1:500, Santa-Cruz, #sc-17342), rabbit anti-S100β (1:500, DAKO, #Z0311), mouse anti-ISL1 (1:250, DSHB, clone 39.4D5-s), rabbit anti-CART (1:500, Phoenix Pharma., #H-003-62), goat anti-CHAT (1:100, Millipore, #AB144P), mouse anti-CD31 (1:200, Dako, clone JC70A), rat anti-CD31 (1:200, BD Pharmingen, #553370), mouse anti-SMA-Cy3 (1:500, Sigma, #C6198), chicken anti-NF200 (1:1000, Abcam, #ab4680).

When DAPI (Sigma, D9542) was used, it was diluted in PBS to a concentration of 0.1 mg/mL and applied on the sections at the same time as the secondary antibodies. For detection of the primary antibodies, secondary antibodies raised in donkey and conjugated with Alexa-488, -555 and -647 fluorophores were used (1:1000, Molecular Probes, Thermo Fisher Scientific).

### Whole Mount Immunofluorescence

Whole mount fluorescent immunostaining of mouse embryos, 3D imaging and visualization were performed as previously described (Adameyko et al., 2012). Briefly, following fixation and PBST washes (see section “Embryo Collection and Preparation”), the samples were progressively dehydrated by incubation at room temperature at 25, 50, 75% and then 100% methanol (MetOH) (1 h incubation per concentration). Then, autofluorescence from the blood was bleached with incubation in Dent’s bleach (one part 30% H_2_O_2_/two parts Dent’s fix) (Dent’s fix: 20% DMSO in 80% MetOH) at 4°C for 24 h. Next, samples were washed in MetOH at room temperature five times, 10 min each and fixed in Dent’s fix for another 24 h at 4°C.

On the day of the staining, the samples were washed in PBST at room temperature three times, 20 min each. Embryos were incubated with the primary antibodies diluted into blocking solution (5% of normal donkey serum, 20% DMSO in PBS) for a week, at room temperature. Following incubation with the primaries, samples were washed in PBST for six times, 30 min each at room temperature and incubated for 2–3 days with secondary antibodies in blocking solution, at room temperature. Upon completion, embryos were washed six times, 30 min each in PBST, dehydrated by incubation for 5 min in 50% MetOH and an hour in 100% MetOH. Prior to imaging, samples were cleared using BABB solution (one part of benzyl alcohol/two parts of benzyl benzoate) for 1 h at room temperature with rotation prior to imaging in BABB.

### Microscopy

Images were acquired using LSM700, LSM 710, LSM 780, LSM 880 Zeiss confocal microscopes equipped with 10x, 20x, 40x, and 63x objectives. Images were acquired in the.lsm format and processed with FIJI (Schindelin et al., 2012).

### Statistics

Data were analyzed using GraphPad Prism (v 7.02) and expressed as mean ± standard error of mean. Statistical significance was calculated by two-sided *t*-tests without assuming a consistent SD and represented as follows: ^∗^*p*-value ≤ 0.05; ^∗∗^*p*-value ≤ 0.01, ^∗∗∗^*p*-value ≤ 0.001. Sample size ranged from 3 to 4 biological replicates.

## Discussion

The ZO is a large accumulation of chromaffin cells, which, unlike the adrenal medulla, is surrounded by sympathetic structures and is lacking an equivalent of the adrenal cortex. The organ appears to have an important role during embryonic and very early postnatal development, releasing catecholamines into the circulation and it regresses following birth (West et al., 1953; Schober et al., 2013). However, during gestation, the adrenal gland forms almost simultaneously with the ZO, posing questions about why a secondary source of catecholamines would be necessary. A plausible explanation is that the ZO matures faster than the adrenal gland and is capable of providing an additional source of catecholamines during critical developmental periods. Another possibility is that the ZO is the evolutionary remnant of the first chromaffin cells in *Agnatha*, prior to the emergence of the mesodermal adrenal cortex, which surrounds the adrenal chromaffin cells.

Importantly, the co-existence of the sympathetic para-ganglia and the chromaffin ZO in such proximity without a clear spatial separation, as in the case of the adrenal medulla and the suprarenal ganglion, pointed to a potential common origin of the two components. For decades, it was believed that sympathetic neurons and chromaffin cells of ZO were derived from freely migrating neural crest cells, which migrate around the dorsal aorta and acquire their differential identity as a response to signals secreted from the dorsal aorta (Huber et al., 2009; Saito et al., 2012). However, some more recent data pointed to an alternative mechanism: the progenitors of the SA lineage are heterogeneous prior to final steps of migration and express different markers before they reach the SA anlage (Ernsberger et al., 2005; Chan et al., 2016). This knowledge points to the existence of at least two lineages of progenitors giving rise to sympathetic and chromaffin cells respectively.

Numerous genes have been found dispensable for normal development of the SA system (Huber, 2006). Such an example is the tyrosine kinase receptor RET, which is expressed by SA progenitors at early embryonic stages and is later downregulated in chromaffin cells while persisting in sympathetic neurons (Pachnis et al., 1993; Allmendinger et al., 2003). Additionally, *Ret* knock-out mice exhibit defective migration and neurite elongation of sympathetic neurons but no defect in chromaffin cell numbers or ultrastructure (Enomoto et al., 2001; Allmendinger et al., 2003). In accordance to these observations, by using the *Ret*^CreERT2^;*R26*^TOM^ strain and injecting TAM at E10.5 and E11.5, 24- and 48-h prior to the detection for the first chromaffin cells in the SA primordium, we showed that at E15.5 *Ret*^TOM^ was expressed specifically in the sympathetic ganglia and was almost absent in the ZO chromaffin cells, suggesting a lineage split of SA progenitors as early as E10.5. This urged us to investigate more genes that have been involved in the SA progenitor lineage. A gene that is indispensable for both sympathoblasts and chromaffin cells is *Ascl1* (*Mash1*) (Huber et al., 2002a). Next, using the inducible *Ascl1*^CreERT2^;*R26*^TOM^ strain, we observed that recombination at E11.5 resulted in *Ascl1*^TOM+^ cells found only in the ZO. Thus, our results showed an established lineage separation between sympathetic and chromaffin progenitors at around E10.5 and E11.5, a time point at which only SOX10^+^ cells are detected at the adrenal gland anlage but no TH^+^ cells (Lumb et al., 2018). At those stages, neural crest cells have completed their migration and no longer exist as freely migrating (Furlan et al., 2017), pointing to an alternative progenitor that is not a neural crest cell.

In line with this, recent studies have brought forward a new type of multipotent cells, the nerve-associated Schwann cell precursors (SCPs). SCPs are multipotent cells that are able to generate a variety of cell types during development, ranging from melanocytes, medullary chromaffin cells and parasympathetic neurons, glomus cells of the carotid organ, enteric neurons and even endoneural fibroblasts, Schwann cells and some mesenchymal populations (Jessen and Mirsky, 2005; Jessen et al., 2008; Adameyko et al., 2009; Laranjeira et al., 2011; Dyachuk et al., 2014; Espinosa-Medina et al., 2014; Isern et al., 2014; Kaukua et al., 2014; Uesaka et al., 2015; Furlan et al., 2017; Hockman et al., 2018). Thus, SCPs use the peripheral nerves to reach specific locations in the developing embryo, which is continuously growing in size and, in a way, are able to functionally replace neural crest cells at late time points of development (Furlan and Adameyko, 2018).

In agreement with these studies, our results showed a new SCP-contribution to ZO, as well as to a small fraction of posterior-trunk sympathoblasts. Lineage tracing induced in *Plp1*^CreERT2^;*R26*^YFP^ embryos at E11.5, a time when no neural crest cells are found anymore except at the tip of the tail, revealed recombination of roughly half the chromaffin cells of the ZO, while no tracing was observed in most sympathetic structures such as the suprarenal ganglion, the mesenteric ganglion and anterior part of the sympathetic chain. Unexpectedly, we also noticed recombination in a small fraction of posterior sympathetic ganglia (around 10% of all sympathoblasts posteriorly to the inferior mesenteric artery). This observation shows that there is a mixed origin of the sympathetic neurons at the posterior part of the body, which was never described so far, since common belief was that all sympathetic neurons are neural-crest-derived. Even though the evolutionary or functional reason for this adaptation is not clear, we hypothesize that it may provide some additional heterogeneity in the sympathetic compartment. To obtain further confirmation of the SCP involvement in ZO development, we injected TAM at E11.5 and E12.5 in mice of the *Sox10*^CreERT2^;*R26*^DTA^ strain and analyzed them at a much later developmental stage (E17.5). This genetic manipulation allowed for almost total glial ablation, as seen by the numbers of SOX10^+^ cells and resulted in a significant decrease in the total chromaffin cell numbers of the ZO, but left all sympathetic ganglia unaffected. However, this is not surprising, when taking in consideration the low proportion of sympathoblasts that are derived from the *Plp1*^+^ lineage. Targeted SCP-ablation might be compensated in the sympathetic component through increased proliferation of the remaining neuroblasts during development.

*Ascl1* is a key transcription factor that is necessary for the development of both sympathetic and chromaffin cells (Huber et al., 2002a). *Ascl1* is considered to be on top of the gene hierarchy that controls the SA cell fate acquisition, expressed by sympathetic and parasympathetic autonomic neurons, as well as, chromaffin cells (Lo et al., 1991; Guillemot and Joyner, 1993) and is necessary for the initiation of *Th* and *Dbh* expression (Hirsch et al., 1998; Lo et al., 1998). Taken together, these studies suggest that *Ascl1* ablation should lead to the accumulation of defective progenitors of the SA fates that are not able to differentiate into *Th*-expressing cells. In that case, we should be able to detect multiple *Ascl1*^+^ SCPs in the ZO anlage. To examine this hypothesis, we used the targeted CreERT2 knock-in strain *Ascl1*^CreERT2^;*R26*^TOM^, which allows the study of the fate of mutant *Ascl1*^-/-^ cells. Indeed, in *Ascl1*^CreERT2/CreERT2^;*R26*^TOM/+^ mutant embryos at E15.5, following TAM injection at E10.5, we observed a plethora of glial-marker-expressing *Ascl1*^TOM+^ cells that were in much fewer numbers in the control *Ascl1*^CreERT2/+^;*R26*^TOM/+^ embryos, accompanied by a significant decrease in TH+ cell numbers. Moreover, we observed an increase of CART^+^/*Ascl1*^TOM+^ cells in the ZO, even though sympathetic ganglia were completely absent in the mutant. We hypothesize that these cells represent an intermediate transition state between the SCPs and fully differentiated CART^-^/TH^+^ chromaffin cells. If this holds true, it would give an interesting insight into the evolutionary emergence of chromaffin cells from sympathetic cells that were found dispersed in the body.

Next, we wondered how SCPs reach the developing ZO primordium. Early on during embryonic development, chromaffin cells and sympathetic neurons receive innervation from the cholinergic preganglionic neurons of the intermediolateral (IML) column of the spinal cord gray matter. We thus hypothesized, that these nerves could serve as the delivery mechanism of the SCPs to the primordium of the forming ZO. To test this, we used the transgenic strain *Hb9*^Cre^;*Isl2*^DTA^ to ablate the neurons of the IML. Nerve ablation resulted in significant chromaffin cell loss in the ZO and a mild phenotype in the posterior sympathetic ganglia, indicating that at least some component of the ZO must also be neural-crest derived or that some of the SCPs reached the forming ZO just before the nerve underwent apoptosis. Moreover, we observed that the ZO was composed from CART^-^/TH^+^ cells and CART^+^/TH^+^ cells, and that both subtypes were significantly reduced upon the visceral nerve ablation. This observation points to the composite nature of the ZO itself, an idea which has been hinted in the past but only due to its association with the sympathetic ganglia around the dorsal aorta. The heterogeneity of the cells composing the ZO might be what makes it differ from the adrenal medulla and could explain its transient role during embryonic development, since a mixture of sympathetic and chromaffin cells could operate as a functional unit of extensive catecholamine and neuropeptide release.

Chromaffin cells and sympathetic neurons share *Th* and *Dbh* expression, genes coding for enzymes that are involved in noradrenaline synthesis. Despite the numerous similarities, it has also been shown that several morphological characteristics and molecular properties define the two cell types (Huber, 2006; Chan et al., 2016; Furlan et al., 2017). The most pronounced characteristics of chromaffin cells that differentiated them from sympathetic neurons was considered the lack of neurites and the presence of dense vesicles filled with catecholamines (also known as chromaffin granules). However, in recent years, technical advancements allowed for single-cell transcriptomic analysis of many cell types and organs, among which are the developing SA system and the postnatal sympathetic ganglia in the mouse (Furlan et al., 2016, 2017). These studies resulted in a large array of data that revealed many more differences between sympathetic neurons and chromaffin cells on the molecular level. However, thus far, these unique markers have not been used to obtain new information about the placement of ZO and associated sympathetic ganglia. In an attempt to examine their organization in space, we performed an array of experiments on whole embryo trunks during early stages of SA development, ranging from E12.5 to E13.5. These clarified the relative position of the mesenteric ganglia and ZO, as well as some TH^+^ cells in the vicinity that have not been described thus far, showing even more clearly the complexity of sympathetic ganglia and extra-adrenal chromaffin bodies. Additionally, we were able to detect a thus far unknown chromaffin body, which we decide to name “transitional chromaffin body” (TCB). As the name suggests, TCB is a small group of TH^high^ cells that is found at the transition from the SRG toward the mesenteric sympathetic ganglion at E15.5 embryos. The cells comprising the TCB were also SCP-derived, as showed *Plp1*^YFP+^ tracing from E11.5. Additionally, the 3D analysis of the area revealed previously undescribed extra-adrenal chromaffin populations in close association with the kidneys. Thus, we show that there are more extra-adrenal chromaffin bodies than those which have been described so far. Furthermore, our 3D analysis of the developing trunk along the antero-posterior axis revealed a mix of chromaffin and sympathetic-like cells in the ZO and the clear separation between the ZO and the sympathetic mesenteric ganglion, despite the proximity and simultaneous development of the two structures. Thus, the ZO is found constantly in the vicinity of sympathetic neurons, in contrast to the adrenal medulla, which is well separated from the sympathetic suprarenal ganglion by means of the adrenal cortex. The importance of sympathetic neurons and chromaffin cells and the ability to differentiate with precision between the two lies in their association with special cancer types found in the clinics, namely PGG and PCC. Currently, these quite diverse cancer types are classified as such based on their location and what seems like their origin at the time of diagnosis. Specifically, PCCs are tumors that are found associated with the adrenal gland, while PGGs are extra-adrenal tumors (Lloyd et al., 2017). PGGs (also referred to as “extra-adrenal pheochromocytoma”) are usually found in the vicinity of the kidneys, even though many other “atypical” locations of such tumors have been described such as peri-adrenal and para-aortic. Apart from these, numerous cases of ZO-associated PCCs or PGGs have been described, making even clearer the need of new classification methods using specific markers rather than just the location of the tumor (Altergott et al., 1985; Martucci and Pacak, 2014). Surprisingly, the diagnostic markers for both PCC and PGG are in many cases common and recent approaches for the molecular classification of these tumors are considering both types as one entity (Fishbein et al., 2017). However, the differences between the sympathetic and chromaffin lineages are not negligible and such approaches that consider the PGG- and PCC-composing cells similar might not be correct. Until recently, due to the notion that all these structures (the ZO, adrenal medulla and sympathetic neurons), were derived from neural crest cells, these cancer types were thought to be originating from a common progenitor. However, our study, combined with others showed that most chromaffin cells in the body are derived from SCPs, pointing to the fact that SCPs can be one of the initiating tumorigenic cell types (Furlan et al., 2017; Lumb et al., 2018). Additionally, we show that the cells composing the adrenal medulla and ZO have the same origin but might be, in fact, quite different. Thus, the tumor-initiating cells could be SCPs, chromaffin cells, sympathetic-like chromaffin cells or sympathoblasts. In the future, proper classification of any cancer type associated either with sympathetic or chromaffin lineages may allow more selective and more effective treatment.

## Conclusion

We focused on the comparative study of the development of the extra-adrenal chromaffin ZO and the surrounding sympathetic ganglia. By employing a variety of complementary approaches, we show for the first time, that the chromaffin cells of the ZO are SCP-derived, in contrast to the majority of the sympathetic neurons of the trunk. Unexpectedly, we also observed that a minority of sympathetic neurons around the posterior-most level of the dorsal aorta is SCP-derived. Our results, in combination with previous studies, suggest that the generation of neuroendocrine cells from SCPs might be favored during the evolution of vertebrates, probably, enabling larger embryos and faster growth rates.

## Author Contributions

MK collected and analyzed the data of Figures 1–3, 5, 6. PK, DK, and VD collected and analyzed the data of Figure 4. MH performed sample collection and processing. AF, FM, UM, FL, SH, and LC-E provided transgenic mouse material. MK composed all figures. MK and IA wrote the manuscript. MK, VD, AF, PE, KF, and IA designed the study. All authors provided scientific input and suggestions prior to manuscript submission.

## Conflict of Interest Statement

The authors declare that the research was conducted in the absence of any commercial or financial relationships that could be construed as a potential conflict of interest.
